# Optimizing Suicide Prevention Programs and Their Implementation in Europe (OSPI Europe): an evidence-based multi-level approach

**DOI:** 10.1186/1471-2458-9-428

**Published:** 2009-11-23

**Authors:** Ulrich Hegerl, Lisa Wittenburg, Ella Arensman, Chantal Van Audenhove, James C Coyne, David McDaid, Christina M van der Feltz-Cornelis, Ricardo Gusmão, Mária Kopp, Margaret Maxwell, Ullrich Meise, Saska Roskar, Marco Sarchiapone, Armin Schmidtke, Airi Värnik, Anke Bramesfeld

**Affiliations:** 1 University of Leipzig, Department of Psychiatry, Germany; 2 Estonian-Swedish Mental Health and Suicidology Institute, Estonia; 3 London School of Economics, Personal Social Services Research Unit, LSE Health and Social Care, UK; 4 Katholieke Universiteit Leuven, LUCAS, Belgium; 5 Trimbos-Instituut/Netherlands Institute of Mental Health and Addiction, The Netherlands; 6 University of Stirling, UK; 7 National Suicide Research Foundation, Ireland; 8 Semmelweis University Budapest, Institute of Behavioural Sciences, Hungary; 9 Institut za varovanje zdravja RS, Slovenia; 10 Bayerische Julius-Maximilians-Universität Würzburg, Department of Clinical Psychology, Clinic for Psychiatry and Psychotherapy, Germany; 11 CEDOC, Departamento de Saúde Mental, Faculdade de Ciências, Médicas da Universidade Nova de Lisboa, Portugal; 12 University of Primorska, PINT, Slovenia; 13 Gesellschaft für Psychische Gesundheit - pro mente tirol, Austria; 14 VU University Medical Centre Institute of Extramural Research, Dept. of Psychiatry, Amsterdam, the Netherlands; 15 University of Pennsylvania, School of Medicine, Pennsylvania, USA; 16 Current address: University of Molise, Health Science Department, Italy

## Abstract

**Background:**

Suicide and non-fatal suicidal behaviour are significant public health issues in Europe requiring effective preventive interventions. However, the evidence for effective preventive strategies is scarce. The protocol of a European research project to develop an optimized evidence based program for suicide prevention is presented.

**Method:**

The groundwork for this research has been established by a regional community based intervention for suicide prevention that focuses on improving awareness and care for depression performed within the European Alliance Against Depression (EAAD). The EAAD intervention consists of (1) training sessions and practice support for primary care physicians,(2) public relations activities and mass media campaigns, (3) training sessions for community facilitators who serve as gatekeepers for depressed and suicidal persons in the community and treatment and (4) outreach and support for high risk and self-help groups (e.g. helplines). The intervention has been shown to be effective in reducing suicidal behaviour in an earlier study, the Nuremberg Alliance Against Depression. In the context of the current research project described in this paper (OSPI-Europe) the EAAD model is enhanced by other evidence based interventions and implemented simultaneously and in standardised way in four regions in Ireland, Portugal, Hungary and Germany.

The enhanced intervention will be evaluated using a prospective controlled design with the primary outcomes being composite suicidal acts (fatal and non-fatal), and with intermediate outcomes being the effect of training programs, changes in public attitudes, guideline-consistent media reporting. In addition an analysis of the economic costs and consequences will be undertaken, while a process evaluation will monitor implementation of the interventions within the different regions with varying organisational and healthcare contexts.

**Discussion:**

This multi-centre research seeks to overcome major challenges of field research in suicide prevention. It pools data from four European regions, considerably increasing the study sample, which will be close to one million. In addition, the study will gather important information concerning the potential to transfer this multilevel program to other health care systems. The results of this research will provide a basis for developing an evidence-based, efficient concept for suicide prevention for EU-member states.

## Background

### Public health relevance of suicide in the European Union

Fatal and non-fatal suicide acts are a significant public health issue. This is especially the case in Europe where the highest rates for completed suicide in the world are found [1]. Every year more than 58,000 persons die by suicide within the European Union. According to World Health Organization (WHO data), suicide is among the 10 leading causes of death for all ages [2]. Suicidal acts pose a considerable burden of disease and death. Suicide needs to be viewed not only as the premature end of a human life but as affecting and traumatising family members and other persons involved. Therefore, in 1984 the WHO's European Member States defined the reduction of suicide as one of its main health policy targets and reinforced this target in several position papers [3]. In addition, preventing suicide is one of the five areas of priority of the European Pact for Mental Health and Well-Being, which was launched by the European Commission in 2008 [4].

Closely related to completed suicides are non-fatal suicidal acts. The rate of non-fatal suicidal acts is estimated to be about 10 times higher than that of suicides. Non-fatal suicidal acts are the strongest predictor for completed suicide, especially in males [5]. Thus, every global strategy to prevent suicide should also include the prevention of non-fatal suicidal acts, not only as a goal in itself, but as an efficient means of preventing completed suicides.

Rates of non-fatal suicidal acts and suicides depend on many factors. It is estimated that in Europe 90% of suicides occur within the context of a psychiatric disorder [6]. Depression is most commonly associated with suicide, but other affective disorders, alcohol and substance abuse disorders, and schizophrenia also frequently underlie suicidal behaviour [7-10]. Yet, suicidality and the transition from non-lethal to lethal acts are complex phenomena, complicated by access to lethal means, gender, and cultural and social factors including attitudes towards suicidality, as well as personality factors such as impulsivity. Because of the multi-factorial nature of suicidality, interventions that address the problem on multiple levels are considered to be most effective. However, strong evidence delineating the most effective strategy to prevent suicidality and the necessary components of suicide prevention programs is lacking [6,11].

In the face of the complexity of risk for suicide, and scarce evidence but nevertheless a high need for on effective suicide prevention on a community level, the European Commission recently committed to financing a research project within the seventh Framework Program with the goal of optimizing suicide prevention programs and their implementation in Europe (OSPI-Europe).

The aim of OSPI-Europe is to provide diverse regional policy makers and the European Commission with an evidence based, efficient concept for suicide prevention along with the corresponding materials and instruments for the multifaceted intervention and guidelines for the implementation process.

The specific study objectives include:

1. To develop a state of the art intervention concept for the prevention of suicidality that considers current evidence based best practices and international experiences with multilevel interventions.

2. To implement a multilevel community based intervention in four culturally different European model regions in a comparable manner.

3. To evaluate the intervention in a controlled, cross-country comparable design including primary and secondary outcomes, as well as to perform process and economic evaluations.

**Figure 1:**
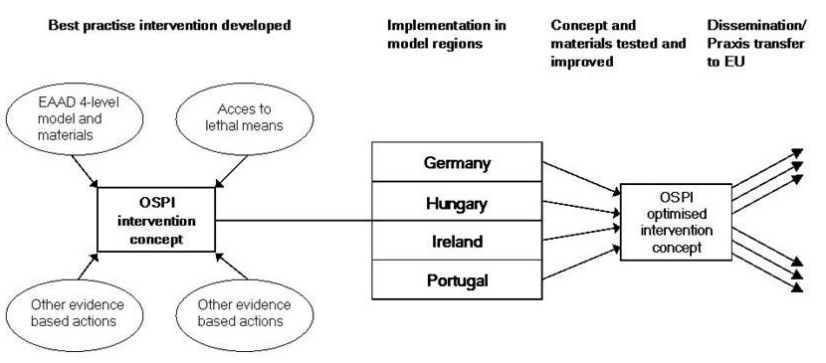
**OSPI-Europe Research Concept**.

An outline of the research concept is presented in Figure 1.

### Groundwork

The groundwork for the OSPI-Europe project was established through a community based multifaceted intervention program for improving care of depression and for preventing suicidality that was implemented in Nuremberg, Germany, a city with a population of 500,000 inhabitants (Nuremberg Alliance Against Depression, NAD) [12]. The Nuremberg intervention approached the prevention of suicide and non-fatal suicidal acts by focusing not only on improving the treatment of depression but also by implementing other measures, such as influencing the media to report suicide in a responsible, non-sensational and respectful manner. The hypothesis was that improvement in depression awareness and treatment would lead to a reduction in suicidal behaviour on a population basis. The NAD comprised a 4-level intervention:

1. Training and practice support for primary care physicians in detecting and treating depression

2. Public relations activities for informing the general public about depression, including anti-stigma campaigns.

3. Training sessions on depression and suicidality for community facilitators such as priests, social workers, geriatric care givers, teachers and journalists who are gatekeepers in a position to direct vulnerable and high risk persons into effective treatment

4. Overtures to high risk groups (persons after non-fatal suicidal acts), establishment of help lines and support of self-help activities involving patients and relatives

The NAD was rigorously evaluated according to a controlled pre-post design with the number of suicidal acts (fatal and non-fatal) as primary outcome. After two years of intervention, a major reduction in the number of suicidal acts was found compared to the baseline year (-24%, p < 0.005). This reduction was clearly significant compared to corresponding changes in the control region (Wuerzburg). The reduction was even more pronounced (-53%, p < 0.01) in secondary analyses examining only the five most lethal suicide attempt methods [13].

The 4-level intervention concept of NAD was further refined and transferred to other EU countries. Thereby the European Alliance Against Depression (EAAD) came together [14-16]. The EAAD was an EU-funded network of partners from 17 countries that all aimed to implement the 4-level intervention concept in their regions, adapting it to local conditions even while preserving what were viewed as the key components. The strong evidence base, materials, concepts and evaluation tools of the NAD, combined with the network and experience of the EAAD constitutes the background, as well as the starting point from which OSPI-Europe was developed.

### Methodological challenges in assessing the effectiveness of suicide prevention programs

One of the challenges of field research on suicide prevention is that because of a low base rate in completed suicides, it has been notoriously difficult to provide strong evidence concerning suicide-preventive effectiveness for any particular measure (e.g. interventions with high risk groups, public relations campaigns, etc) [11]. The smaller the population under observation, the higher the risk to miss statistically even highly relevant effects on suicide rates. In order to address this issue OSPI-Europe seeks to increase statistical power by two approaches: (1) Increasing the size of the population under observation (denominator) by aggregating data from four regions that implement a similar suicide prevention program, and (2) increasing the numerator by constructing a composite primary outcome, consisting of completed suicides but also non-fatal suicidal acts.

## Methods/design

### Development of a state of the art intervention concept

Prior to designing the optimal intervention, the literature was been systematically reviewed and experts in suicidality research consulted. The criterion for selecting individual preventive strategies was scientific evidence for4 effectiveness. In addition to evaluating the effects of the five intervention levels separately, the synergistic effects of the multifaceted approach will also be taken into account. The target population, in which suicide is to be prevented, ranges from adolescence to old age (high rate of suicide in most countries).

A core element of the state of the art intervention developed through OSPI-Europe is the EAAD/NAD 4-level intervention concept and the available materials which are already in use within the EAAD. There is lower level evidence available that the individual strategies combined within the 4-level intervention concept may be effective [6,11]. However, strong evidence is available showing that the four-level intervention is effective as a package [13]. In the context of the research presented here, the original EAAD four-level intervention is enhanced by the inclusion of additional strategies that have recently demonstrated a potential for efficacy. Thus, as part of the optimisation process, efforts to restrict access to lethal means will comprise a fifth intervention level of OSPI-Europe. The main focus of the fifth level will include actions such as working with local councils to install higher railings at bridges and encouraging the prescription of antidepressants that are less likely to be lethal in overdoses.

### Implementation of a multilevel community based intervention

The intervention concept developed will be implemented and tested in regions of four countries (Ireland, Germany, Hungary, Portugal) in a comparable manner. The countries were chosen to represent different EU-health systems and different socio-cultural characteristics as outlined in Table 1.

**Table 1:** Main characteristics of intervention countries and intervention and control regions

*Country*	*Health System*	*intervention region*	*control region*
*Hungary*	Centralised national health insurance fund, limited private sector	Miskolc population 180,000	Szeged population 170,000
*Ireland*	Tax funded public health service, with supplemental voluntary insurance	Limerick population 184,055	Galway population 231,670
*Portugal*	mix of National Health Service, special social health insurance schemes for certain professions and private health insurance	Amadora population 200,000	Almada population 110,000
*Germany*	Bismarckian/social health insurance system with strong public-private partnership	Leipzig population 500,000	Magdeburg population 230,000

It was considered unrealistic to randomly select intervention regions from all EU member states because of the multiple factors on which representativeness could be called into question.

Within each of the four model countries, intervention regions are established. Interventions are implemented in a standardised and synchronized way to ensure comparability across the regions. This means that interventions contain the same core elements (defined by minimum standard of implementation type and intensity) that are defined as "obligatory interventions" and are implemented in a similar time frame, and in each of the four regions. In addition "optional interventions" are also defined. These can be implemented in the regions according to local requirements and resources. By distinguishing between obligatory and optional interventions some modification and adaptation to local needs in the different regions is allowed, but the basic concept is preserved across intervention sites. The intervention will be implemented in the four regions over a period of at least 18 months, starting in 2009.

### Evaluation of the intervention

The intervention is evaluated according to a prospective and controlled design. Therefore control regions are chosen in each intervention country. Control regions are comparable to intervention regions in terms of urbanity (please see Table 1 for further details). Evaluations will be performed based on primary and intermediate outcomes. Additionally, a process evaluation and an evaluation of the economic costs and consequences of the intervention will be conducted.

#### Primary and secondary outcomes

The **primary outcome **for evaluating the effect of the intervention is the rate of suicidal acts (fatal + non-fatal) in the intervention and the control regions at baseline (six months prior to the start of the intervention), during the 18 months of the intervention and six months after. The main hypothesis is that the number of suicidal acts will decrease in each intervention region compared to baseline (six months prior to the start of the intervention) and that this decrease is significantly stronger than changes observed in the corresponding control regions.

For the rates of completed suicides in intervention and control regions, the rates published by the respective statistical offices are used. Data on non-fatal suicidal acts are collected in general hospital emergency rooms, where persons after non-fatal suicidal acts seek medical help. Information on non-fatal suicidal acts is recorded using the instrument of the Monitoring Suicidal Behaviour in Europe (MONSUE) project which is based on the WHO/Euro Multicentre Study on Suicidal Behaviour [17].

#### Intermediate outcomes

In order to further measure the effectiveness of the OSPI-Europe intervention, the study aims to assess intermediate outcomes associated with the single interventions that make up the OSPI-Europe intervention package. These foci on more short term effects are directly linked to the operational goals and the content of the interventions, such as improved awareness, knowledge, confidence, and attitude change.

Prior to the baseline assessment in the four participating intervention regions, a standard evaluation methodology for intermediate outcome criteria is developed based on review of the relevant literature, a review of the existing EAAD evaluation catalogue and consultation with EAAD I and EAAD II partners, as well other researchers/experts in suicide prevention.

The evaluation of intermediate outcomes includes the following factors:

##### Public attitudes

A population survey on attitudes and knowledge towards depression and suicidality is performed in the intervention and control regions at baseline and about 12 months following intervention implementation. The survey is based on existing validated instruments: the Depression Stigma Scale (DSS), [18] and the Attitude Toward Seeking Professional Psychological Help (ATSPPH-SF) questionnaire [19]. Five hundred people sampled from the general population will complete baseline and follow-up telephone interviews in all intervention and control regions. These will be conducted by a single independent survey company to ensure consistency of implementation across all regions.

##### Treatment of depression

As a proxy for the treatment of depression, prescription rates for antidepressants and other psychopharmaceuticals will be monitored in the intervention regions by using health insurance data or equivalents, available in the intervention countries.

##### Training programs

Effects of training for community facilitators and general practitioners will be evaluated. Evaluation will be based on questionnaires such as the DSS [18], Suicide Intervention Response Inventory (SIRI-2) [20], confidence scales [21,22], intervention knowledge test [23], Depression Attitude Questionnaire (DAQ) [24,25] and the Attitude Toward Suicide Prevention Scale [26]. Questionnaires will be tailored to the specific target groups where necessary. In addition, general practitioners' referral to psychological treatment will be evaluated, before and after trainings, as well as at four months follow-up.

##### Media reporting

Media reports on suicide (such as articles in the local newspapers) will be collected in the intervention and control regions. The number (quantitative assessment) and the content of the reporting (qualitative analyses) will be compared before and after the intervention. The desired outcome is media reporting that is in line with WHO and IASP guidelines for responsible suicide reporting.

#### Economic evaluation

It is important in a situation where resources are limited to consider not only if something works, and in what context, but also at what costs. Economic evaluation can help aid decision makers address this question. It compares the costs and effectiveness of two or more interventions. Our hypothesis is that the intervention will lead to reductions in the numbers of completed suicides and non-fatal suicidal acts; this in turn will be associated with an overall reduction in the use of resources, such as health care and emergency services.

In order to calculate the economic costs and consequences of the interventions, firstly the costs of suicide and non-fatal suicidal acts will be estimated in each of the four countries. This will be done from both a public purse and societal perspective. A questionnaire will be developed to collect information on the typical resource use for suicidal events, for instance police time inputs and the need for ongoing treatment and support for non-fatal suicidal acts. Lifetime productivity losses to the economy due to premature mortality will be valued using age adjusted average wage rates. Intangible costs associated with suicide such as the pain and shock experienced by family members will be imputed using data on the economic impacts of sudden death from road traffic accidents. These data have previously been used in valuing the costs of suicide in Ireland and Scotland [27,28].

Secondly, resources, including unpaid volunteer inputs, required to deliver the intervention will be collated using expert responses to a modified version of the Client Service Receipt Inventory (CSRI) [29]; in addition, this instrument will also gather data on some of the costs associated with the initial development and implementation of the OSPI-Europe intervention.

Using this data we will then be able to construct a cost effectiveness analysis comparing changes in the rate of suicidal acts in the intervention and control areas with the costs of delivering the interventions less the costs avoided as a result of suicides averted. Sensitivity analysis will be used to test how varying assumptions on costs, effectiveness and fidelity of implementation impact on the likelihood that the intervention will be cost effective. This will be demonstrated visually using cost effectiveness acceptability curves. Finally we will also use decision analytical modelling to help project the long term potential costs and consequences of the OSPI-intervention of beyond the duration of our empirical study period.

#### Evaluation of the implementation process

Data about the actual process of implementation will provide valuable information about the obstacles encountered and the fidelity of the implementation in the intervention regions. Specifically at play are the characteristics of the local environment which have the potential to influence both suicidal behaviour and the effective implementation of a prevention program. Key issues to be considered beyond prevailing local attitudes toward mental health treatment (which is assessed also as an intermediate outcome) and patterns of suicidality, concern local health care structures and resources, ongoing local actions or national actions targeting suicidality, and other factors affecting mental well-being such as unemployment rates. This contextual information is gathered at both national and local level and is used to aid comparisons and understanding of differences in outcomes between each pair of intervention and control regions, as well as across the four interventions sites.

In addition, key stakeholders across all four intervention sites will be interviewed at six monthly intervals on the progress in the implementation process using semi-structured interviews, which will be transcribed and translated into English. This will include gathering information on the local context (and any important intervening events) as well the actual implementation process. This includes information on whether the intervention will succeed in involving all necessary stakeholders, information on the activities that are implemented in the region and the resources available. Of particular interest is additional funding/support, that is made available to the local activities, the barriers and facilitators that have impacted on the implementation process and the sustainability of the interventions. The Community Capacity Index (CCI) [30] will be used to assess the added value for regions in engaging in a multi-level intervention and the sustainability of local activities. This will compliment more quantitative outcomes from the OSPI-Europe intervention.

Finally, the number of activities that form part of the intervention such as public events, leaflets distributed, training sessions held and self-help groups founded will be documented in the intervention regions.

### Funding and Consortium

The project is funded by the 7^th ^Research Framework Program of the European Union for the duration of four years. The consortium consists of 14 partners, most of whom took part in EAAD. Therefore, the project will benefit from previously developed working alliances and a culture of discussion and mutual understanding already existing in the EAAD.

### Ethical principles

The study is planned and will be executed in accordance with the principles laid down in the Helsinki declaration (Edinburgh, Scotland amendment, October 2000). The study protocol was approved both by the Data Protection Commission of Saxony, Germany and the Medical Ethical Board of the Leipzig University. In addition the other study centres in Hungary, Ireland and Portugal are currently seeking ethical approval from their local authority.

## Discussion

OSPI-Europe aims to provide the EU-member states with realistic evidence-based recommendations and decision-support regarding the design and implementation of effective programs to reduce suicidality. The concrete focus on implementation allows to study the efficiency of the prevention program, as well as a process evaluation of the implementation itself. Further, the practicability of the developed materials and instruments, possible shortcomings, potentials for improvement will be identified and improvements will be made based on lessons learned. Thereby, the OSPI-Europe project seeks to bridge the gap between theoretical recommendations and practical application.

The limitations of the study design originate in the challenges of simultaneously implementing comparable interventions in four different countries and health systems. However, process evaluation will entail the collection of important information regarding the transferability of the intervention to different health systems. The close monitoring of the implementation process will help to control and explain differences in implementation that might occur at the four intervention sites.

The existence of different health care systems may also complicate evaluation of the intervention. Availability of health services and specialised mental health care, access to psychotherapy, as well as requirements for out of pocket payments for medication and health services differ in the four intervention countries. Data for antidepressant prescriptions are available in some countries as health insurance data (Germany), and in others as IMS (Intercontinental Marketing Services) data. Close cooperation, harmonisation of terms and definitions as well as consenting on minimum standards within the project consortium are needed to address the challenges with simultaneous data collection.

Further challenges emerge by conducting research within real world local environments where it is impossible to control other intervening activities such as the introduction of National Suicide Prevention strategies (which may or may not include specific prevention activities) or other local initiatives on depression. Other intervention factors, such as the current economic downturn, which may, for instance, lead to significant job losses in some study areas, may also blur the effectiveness of the intervention. Monitoring of intermediate effects, as well as precisely documenting environmental conditions prior to and during the study within intervention and control regions, should allow for more robust interpretation and understanding of primary and secondary outcomes.

The strengths of the design relate to its being grounded in an intervention and evaluation concept that has previously been shown to be effective [13]. Moreover, the European Alliance Against Depression (EAAD) has demonstrated that it is possible to implement basic elements of the OSPI-Europe intervention in different European countries and health systems according to a standardised procedure [15]. Finally, the OSPI-Europe consortium is built on the foundations of the EAAD, and thus draws on the networks, expertise, prior collaboration and knowledge exchange culture that has been developed in the different EAAD countries and during EAAD co-operations since its foundation in 2004.

For OSPI-Europe to make a contribution to suicide prevention in the European Community, OSPI-Europe needs not only to construct a strong intervention and conduct high level research, but it also needs to disseminate results effectively. Dissemination strategies, which are not presented in detail in the context of this paper, remain a key component of the project. Strategies tailored to different audiences can help facilitate the translation of research results into policies and action. This article is meant to be a contribution to this dissemination strategy.

## Competing interests

The authors declare that they have no competing interests.

## Authors' contributions

UH is principle investigator, all participants have contributed their part of expertise and read and approved the latest version of the article.

## Pre-publication history

The pre-publication history for this paper can be accessed here:

http://www.biomedcentral.com/1471-2458/9/428/prepub
